# *trans*-Bromido(pyrimidinyl-κ*C* ^2^)bis­(triphenyl­phosphane-κ*P*)palladium(II)

**DOI:** 10.1107/S1600536810038511

**Published:** 2010-10-02

**Authors:** Gene-Hsiang Lee, Hsiao-Fen Wang, Kuang-Hway Yih

**Affiliations:** a Instrumentation Center, College of Science, National Taiwan University, Taipei 106, Taiwan; b Department of Applied Cosmetology, Hungkuang University, Shalu 433, Taichung, Taiwan

## Abstract

In the title complex, [PdBr(C_4_H_3_N_2_)(C_18_H_15_P)_2_], the geometry around the Pd^II^ atom is distorted square-planar with the Pd^II^ atom displaced by 0.0150 (5) Å from the least-squares BrP_2_C plane. Two PPh_3_ ligands are in *trans* positions [P—Pd—P = 176.743 (17)°], while the pyrimidinyl ligand and Br atom are *trans* to one another [C—Pd—Br = 176.56 (5)°]. Structural parameters from NMR, IR and mass spectra are in agreement with the crystal structure of the title compound.

## Related literature

For reactions in organic synthesis that form C—C bonds, see: Steffen *et al.* (2005 ▶); Beeby *et al.* (2004 ▶); Chin *et al.* (1988 ▶); Dobrzynski & Angelici (1975 ▶). For Pd—C(carbene) bond lengths, see: Cardin *et al.* (1972 ▶) and for Pd—Br bond lengths, see: Yih & Lee (2008 ▶); Yih *et al.* (2009 ▶). For 4,6-dimethyl-2-mercaptopyrimidine, see: Hong *et al.* (2002 ▶).
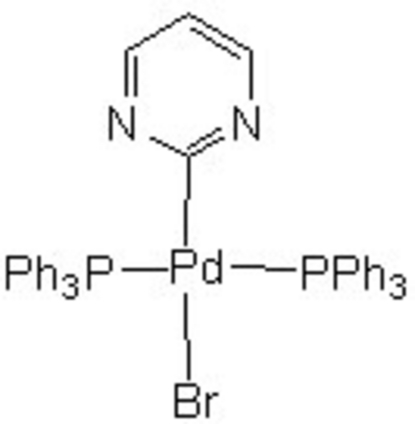


## Experimental

### 

#### Crystal data

[PdBr(C_4_H_3_N_2_)(C_18_H_15_P)_2_]
*M* *_r_* = 789.93
Triclinic, 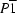

*a* = 12.1051 (8) Å
*b* = 12.7791 (8) Å
*c* = 12.8987 (8) Å
α = 90.257 (2)°
β = 117.044 (2)°
γ = 105.580 (2)°
*V* = 1693.11 (19) Å^3^
*Z* = 2
Mo *K*α radiation
μ = 1.86 mm^−1^
*T* = 150 K
0.50 × 0.35 × 0.25 mm

#### Data collection

Bruker SMART APEX CCD area-detector diffractometer
Absorption correction: multi-scan (*SADABS*; Bruker, 2001 ▶) *T*_min_ = 0.457, *T*_max_ = 0.654
22016 measured reflections
7762 independent reflections
7066 reflections with *I* > 2σ(*I*)
*R*_int_ = 0.023

#### Refinement

*R*[*F* ^2^ > 2σ(*F* ^2^)] = 0.023
*wR*(*F* ^2^) = 0.058
*S* = 1.02
7762 reflections
415 parameters
2 restraints
H-atom parameters constrained
Δρ_max_ = 0.40 e Å^−3^
Δρ_min_ = −0.40 e Å^−3^

### 

Data collection: *SMART* (Bruker, 2007 ▶); cell refinement: *SAINT* (Bruker, 2007 ▶); data reduction: *SAINT*; program(s) used to solve structure: *SHELXS97* (Sheldrick, 2008 ▶); program(s) used to refine structure: *SHELXL97* (Sheldrick, 2008 ▶); molecular graphics: *XP* in *SHELXTL* (Sheldrick, 2008 ▶); software used to prepare material for publication: *SHELXTL*.

## Supplementary Material

Crystal structure: contains datablocks I, global. DOI: 10.1107/S1600536810038511/jh2211sup1.cif

Structure factors: contains datablocks I. DOI: 10.1107/S1600536810038511/jh2211Isup2.hkl

Additional supplementary materials:  crystallographic information; 3D view; checkCIF report

## Figures and Tables

**Fig. 1.**
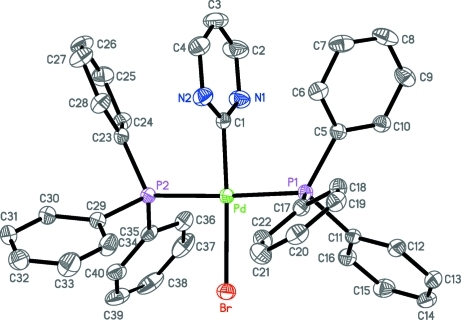
The molecular structure of title compound, with atom labels and 50% probability displacement ellipsoids for non-H atoms.

## supplementary crystallographic information

### Comment

C—C coupling reactions of pyrimidinyl nickel complexes (Steffen *et al.*, 2005), Suzuki cross-coupling reactions of pyridyl-bridged palladium complex (Beeby, *et al.*, 2004), and intramolecular reductive elimination of Pd—N binuclear complex [Pd(µ-C_9_H_6_N)(µ-dppm)]_2_(Cl)_2_ (Chin *et al.*, 1988) are some of important reactions in organic synthesis by forming C—C bond (Dobrzynski & Angelici, 1975). To our knowledge, no 2-palladiumpyrimidine crystal structure has been described.

To synthesis of 2-palladiumpyrimidine compound, complex [Pd(PPh_3_)_4_] was used to react with 2-bromopyrimidine in dichloromethane at room temperature. As a result, a two triphenylphosphine displaced complex [Pd(Br)(C_4_H_3_N_2_)(PPh_3_)_2_] was isolated with 95% yield. The X-ray crystal structure analysis has been carried out to provide structural parameters.

The molecular structure of the title compound is shown in Fig. 1. In the title complex (I), the palladium atom has a distorted square planar geometry. The palladium atom is displaced by 0.0150 (5)Å from the least-squares plane of BrP1P2C1. The Pd—C1 bond distance, 1.9985 (18) Å, is longer than other Pd^II^-carbon(carbonyl) distances, and similar to those of Pd—C(carbene) distances (Cardin *et al.*, 1972, and references therein). Two PPh_3_ ligands are in *trans* position: P1—Pd—P2, 176.743 (17)°, while the pyrimidinyl ligand and bromide are *trans* to each other: C1—Pd—Br1, 176.56 (5)°. The Pd—N bond distances (2.8489 (17) and 2.8703 (16) Å) indicate no bonding interaction between the nitrogen atom and palladium metal atom. Within the pyrimidinyl ligand itself, the geometry is consistent with a significant partial double bond character in the C—C and C—N bond. The C—N bond distances (1.330 (2) ~1.340 (3) Å) are typical for a C—N bond having partial double bond character and are certainly much shorter than the normal C—N (1.47 Å) single bond. The Pd—C1 (1.9985 (18) Å) and Pd—Br (2.5353 (3) Å) lengths of (I) are in agreement with reported value (Yih *et al.*, 2008, 2009).

The ^31^P{^1^H} NMR spectra of (I) shows a singlet resonances at δ 21.4. In the ^1^H NMR spectra, the 4-H and 5-H protons of the pyrimidinyl group exhibit two singlet resonances at δ 7.86 and at δ 7.52. The ^13^C{^1^H} NMR spectra of (I) reveals two singlet at δ 114.2 and at δ 154.4 which are assigned to the 5-C and 4-C carbon atom of the pyrimidinyl group. It is also noted the IR spectrum of the title complex (I) shows two stretching bands at 1546 and 1537 cm^-1^ for C═N groups. In the FAB mass spectra, base peak with the typical Pd isotope distribution is in agreement with the [*M*^+^] molecular mass of (I).

### Experimental

The synthesis of the title compound (I) was carried out as follows. 2-Bromo-pyrimidine (0.191 g, 1.2 mmol) was added to a flask (100 ml) containing Pd(PPh_3_)_4_ (1.155 g, 1.0 mmol) and CH_2_Cl_2_ (20 ml) at ambient temperature. The mixture was stirred for 2 h. The solvent was concentrated to 10 ml, and 20 ml of diethyl ether was added to the solution. The pale-yellow solids were formed which were isolated by filtration (G4), washed with n-hexane (2 *x* 10 ml) and subsequently dried under vacuum yielding 0.750 g (95%) of the complex [Pd(PPh_3_)_2_(C_4_H_3_N_2_)Br], (I). Spectroscopic data for (I): ^31^P{^1^H} NMR: δ 21.4 (s, PPh_3_). ^1^H NMR: δ 7.23–7.66 (m, 30H, 2PPh_3_), 7.52 (s, 1H, 5-H of pyrimidinyl), 7.86 (s, 2H, 4-H of pyrimidinyl). ^13^C{^1^H} NMR: δ 128.0 (m, *o*-C of Ph), 129.9 (m, *p*-C of Ph), 134.8 (m, *m*-C of Ph), 114.2 (s, 4-C of pyrimidinyl), 154.4 (s, 5-C of pyrimidinyl). MS (FAB, NBA, m/*z*): 789 [*M*^+^]. Anal. Calcd. for C_40_H_33_BrN_2_P_2_Pd: C, 60.82; H, 4.21; N, 3.55. Found: C, 60.94; H, 4.31; N, 3.18.

### Refinement

H atoms were positioned geometrically and refined using a riding model, with C—H = 0.95 Å and with *U*_iso_(H) = 1.2 times *U*_eq_(C).

### Crystal data

**Table:**

[PdBr(C_4_H_3_N_2_)(C_18_H_15_P)_2_]	*Z* = 2
*M**_r_* = 789.93	*F*(000) = 796
Triclinic, *P*1	*D*_x_ = 1.549 Mg m^−^^3^
Hall symbol: -P 1	Mo *K*α radiation, λ = 0.71073 Å
*a* = 12.1051 (8) Å	Cell parameters from 5205 reflections
*b* = 12.7791 (8) Å	θ = 2.2–27.5°
*c* = 12.8987 (8) Å	µ = 1.86 mm^−^^1^
α = 90.257 (2)°	*T* = 150 K
β = 117.044 (2)°	Rod, light yellow
γ = 105.580 (2)°	0.50 × 0.35 × 0.25 mm
*V* = 1693.11 (19) Å^3^	

### Data collection

**Table:**

Bruker SMART APEX CCD area-detector diffractometer	7762 independent reflections
Radiation source: fine-focus sealed tube	7066 reflections with *I* > 2σ(*I*)
graphite	*R*_int_ = 0.023
ω scans	θ_max_ = 27.5°, θ_min_ = 1.9°
Absorption correction: multi-scan (*SADABS*; Bruker, 2001)	*h* = −15→15
*T*_min_ = 0.457, *T*_max_ = 0.654	*k* = −16→16
22016 measured reflections	*l* = −16→16

### Refinement

**Table:**

Refinement on *F*^2^	Primary atom site location: structure-invariant direct methods
Least-squares matrix: full	Secondary atom site location: difference Fourier map
*R*[*F*^2^ > 2σ(*F*^2^)] = 0.023	Hydrogen site location: inferred from neighbouring sites
*wR*(*F*^2^) = 0.058	H-atom parameters constrained
*S* = 1.02	*w* = 1/[σ^2^(*F*_o_^2^) + (0.0252*P*)^2^ + 0.8169*P*] where *P* = (*F*_o_^2^ + 2*F*_c_^2^)/3
7762 reflections	(Δ/σ)_max_ = 0.003
415 parameters	Δρ_max_ = 0.40 e Å^−^^3^
2 restraints	Δρ_min_ = −0.40 e Å^−^^3^

### Special details

**Table:**

Geometry. All e.s.d.'s (except the e.s.d. in the dihedral angle between two l.s. planes) are estimated using the full covariance matrix. The cell e.s.d.'s are taken into account individually in the estimation of e.s.d.'s in distances, angles and torsion angles; correlations between e.s.d.'s in cell parameters are only used when they are defined by crystal symmetry. An approximate (isotropic) treatment of cell e.s.d.'s is used for estimating e.s.d.'s involving l.s. planes.
Refinement. Refinement of *F*^2^ against ALL reflections. The weighted *R*-factor *wR* and goodness of fit *S* are based on *F*^2^, conventional *R*-factors *R* are based on *F*, with *F* set to zero for negative *F*^2^. The threshold expression of *F*^2^ > σ(*F*^2^) is used only for calculating *R*-factors(gt) *etc*. and is not relevant to the choice of reflections for refinement. *R*-factors based on *F*^2^ are statistically about twice as large as those based on *F*, and *R*- factors based on ALL data will be even larger.

### Fractional atomic coordinates and isotropic or equivalent isotropic displacement parameters (Å^2^)

**Table:**

	*x*	*y*	*z*	*U*_iso_*/*U*_eq_	
Pd	0.373889 (12)	0.223763 (11)	0.271056 (12)	0.01685 (4)	
Br	0.402981 (18)	0.428222 (15)	0.282253 (18)	0.02526 (5)	
P1	0.16970 (4)	0.18272 (4)	0.26328 (4)	0.01688 (9)	
P2	0.57904 (4)	0.25675 (4)	0.28544 (4)	0.01794 (9)	
N1	0.31551 (17)	0.01119 (14)	0.15413 (15)	0.0285 (4)	
N2	0.40272 (16)	0.02238 (14)	0.36125 (15)	0.0265 (4)	
C1	0.36051 (17)	0.06436 (15)	0.26102 (16)	0.0195 (3)	
C2	0.3112 (2)	−0.09446 (18)	0.1485 (2)	0.0391 (5)	
H2	0.2787	−0.1355	0.0736	0.047*	
C3	0.3515 (2)	−0.14614 (18)	0.2462 (2)	0.0387 (5)	
H3	0.3476	−0.2213	0.2406	0.046*	
C4	0.3980 (2)	−0.08343 (17)	0.35255 (19)	0.0324 (5)	
H4	0.4277	−0.1162	0.4222	0.039*	
C5	0.06796 (17)	0.03960 (15)	0.21717 (16)	0.0191 (4)	
C6	0.10631 (19)	−0.03726 (16)	0.29124 (17)	0.0245 (4)	
H6	0.1829	−0.0140	0.3651	0.029*	
C7	0.0338 (2)	−0.14719 (17)	0.25813 (19)	0.0298 (4)	
H7	0.0612	−0.1993	0.3086	0.036*	
C8	−0.0787 (2)	−0.18086 (17)	0.1512 (2)	0.0323 (5)	
H8	−0.1295	−0.2560	0.1289	0.039*	
C9	−0.1176 (2)	−0.10562 (16)	0.07663 (18)	0.0284 (4)	
H9	−0.1946	−0.1292	0.0031	0.034*	
C10	−0.04417 (18)	0.00417 (15)	0.10901 (17)	0.0216 (4)	
H10	−0.0705	0.0555	0.0571	0.026*	
C11	0.06149 (17)	0.25643 (14)	0.16714 (16)	0.0190 (4)	
C12	−0.04724 (18)	0.26179 (15)	0.17709 (17)	0.0237 (4)	
H12	−0.0637	0.2294	0.2368	0.028*	
C13	−0.13095 (19)	0.31432 (16)	0.10011 (18)	0.0269 (4)	
H13	−0.2045	0.3179	0.1075	0.032*	
C14	−0.1082 (2)	0.36161 (16)	0.01236 (18)	0.0293 (4)	
H14	−0.1657	0.3977	−0.0401	0.035*	
C15	−0.0016 (2)	0.35582 (17)	0.00179 (18)	0.0295 (4)	
H15	0.0137	0.3873	−0.0589	0.035*	
C16	0.08367 (19)	0.30429 (15)	0.07917 (17)	0.0233 (4)	
H16	0.1576	0.3018	0.0719	0.028*	
C17	0.18063 (17)	0.21461 (15)	0.40637 (16)	0.0198 (4)	
C18	0.08158 (18)	0.15884 (16)	0.43204 (17)	0.0230 (4)	
H18	0.0093	0.1015	0.3757	0.028*	
C19	0.08851 (19)	0.18684 (17)	0.53916 (17)	0.0257 (4)	
H19	0.0212	0.1487	0.5563	0.031*	
C20	0.1937 (2)	0.27055 (17)	0.62123 (17)	0.0275 (4)	
H20	0.1983	0.2897	0.6947	0.033*	
C21	0.2918 (2)	0.32624 (17)	0.59664 (18)	0.0300 (4)	
H21	0.3635	0.3839	0.6530	0.036*	
C22	0.28583 (19)	0.29816 (16)	0.48958 (17)	0.0256 (4)	
H22	0.3539	0.3362	0.4733	0.031*	
C23	0.63295 (17)	0.14058 (15)	0.26504 (17)	0.0205 (4)	
C24	0.63245 (19)	0.10873 (16)	0.16108 (17)	0.0242 (4)	
H24	0.6049	0.1493	0.0974	0.029*	
C25	0.6716 (2)	0.01883 (17)	0.14949 (19)	0.0304 (4)	
H25	0.6705	−0.0020	0.0782	0.036*	
C26	0.7119 (2)	−0.04028 (17)	0.2415 (2)	0.0323 (5)	
H26	0.7402	−0.1011	0.2342	0.039*	
C27	0.7112 (2)	−0.01134 (18)	0.3444 (2)	0.0337 (5)	
H27	0.7370	−0.0534	0.4070	0.040*	
C28	0.67292 (19)	0.07904 (17)	0.35664 (18)	0.0270 (4)	
H28	0.6740	0.0991	0.4282	0.032*	
C29	0.71134 (17)	0.33035 (15)	0.42804 (16)	0.0213 (4)	
C30	0.84045 (18)	0.34118 (16)	0.45735 (18)	0.0260 (4)	
H30	0.8595	0.3091	0.4033	0.031*	
C31	0.9408 (2)	0.39853 (17)	0.56511 (19)	0.0316 (5)	
H31	1.0286	0.4063	0.5843	0.038*	
C32	0.9138 (2)	0.44424 (18)	0.6445 (2)	0.0379 (5)	
H32	0.9828	0.4835	0.7183	0.045*	
C33	0.7862 (2)	0.4330 (2)	0.6167 (2)	0.0397 (5)	
H33	0.7677	0.4645	0.6715	0.048*	
C34	0.6852 (2)	0.37604 (18)	0.50921 (18)	0.0311 (5)	
H34	0.5977	0.3682	0.4909	0.037*	
C35	0.59335 (18)	0.33938 (15)	0.17515 (17)	0.0210 (4)	
C36	0.5034 (2)	0.29949 (17)	0.05672 (18)	0.0282 (4)	
H36	0.4329	0.2344	0.0366	0.034*	
C37	0.5167 (2)	0.3544 (2)	−0.0311 (2)	0.0376 (5)	
H37	0.4576	0.3251	−0.1114	0.045*	
C38	0.6157 (3)	0.4516 (2)	−0.0027 (2)	0.0406 (6)	
H38	0.6243	0.4893	−0.0631	0.049*	
C39	0.7017 (2)	0.49357 (18)	0.1138 (2)	0.0362 (5)	
H39	0.7687	0.5611	0.1333	0.043*	
C40	0.6914 (2)	0.43795 (16)	0.20293 (19)	0.0263 (4)	
H40	0.7514	0.4674	0.2829	0.032*	

### Atomic displacement parameters (Å^2^)

**Table:**

	*U*^11^	*U*^22^	*U*^33^	*U*^12^	*U*^13^	*U*^23^
Pd	0.01563 (7)	0.01653 (7)	0.01959 (7)	0.00490 (5)	0.00931 (6)	0.00247 (5)
Br	0.02570 (10)	0.01863 (9)	0.03430 (11)	0.00637 (7)	0.01675 (9)	0.00424 (8)
P1	0.0163 (2)	0.0174 (2)	0.0180 (2)	0.00520 (17)	0.00902 (18)	0.00283 (17)
P2	0.0165 (2)	0.0182 (2)	0.0203 (2)	0.00569 (17)	0.00951 (18)	0.00209 (18)
N1	0.0332 (9)	0.0225 (8)	0.0243 (9)	0.0072 (7)	0.0099 (7)	0.0001 (7)
N2	0.0291 (9)	0.0254 (8)	0.0253 (8)	0.0116 (7)	0.0114 (7)	0.0066 (7)
C1	0.0158 (8)	0.0193 (7)	0.0242 (9)	0.0059 (7)	0.0099 (7)	0.0045 (7)
C2	0.0500 (14)	0.0248 (11)	0.0315 (12)	0.0091 (10)	0.0115 (10)	−0.0059 (9)
C3	0.0465 (13)	0.0196 (10)	0.0445 (13)	0.0125 (9)	0.0157 (11)	0.0042 (9)
C4	0.0352 (11)	0.0293 (11)	0.0328 (11)	0.0143 (9)	0.0136 (9)	0.0122 (9)
C5	0.0199 (8)	0.0179 (8)	0.0240 (9)	0.0051 (7)	0.0144 (7)	0.0024 (7)
C6	0.0286 (10)	0.0256 (10)	0.0249 (10)	0.0107 (8)	0.0160 (8)	0.0064 (8)
C7	0.0473 (13)	0.0226 (10)	0.0344 (11)	0.0138 (9)	0.0298 (10)	0.0100 (8)
C8	0.0459 (13)	0.0191 (10)	0.0392 (12)	0.0011 (9)	0.0308 (11)	0.0006 (9)
C9	0.0296 (10)	0.0249 (10)	0.0280 (10)	0.0009 (8)	0.0156 (9)	−0.0040 (8)
C10	0.0237 (9)	0.0208 (9)	0.0239 (9)	0.0074 (7)	0.0137 (8)	0.0032 (7)
C11	0.0183 (8)	0.0162 (8)	0.0199 (9)	0.0051 (7)	0.0070 (7)	−0.0008 (7)
C12	0.0240 (9)	0.0227 (9)	0.0265 (10)	0.0085 (8)	0.0127 (8)	0.0033 (8)
C13	0.0222 (9)	0.0234 (10)	0.0342 (11)	0.0093 (8)	0.0113 (8)	−0.0004 (8)
C14	0.0286 (10)	0.0204 (9)	0.0316 (11)	0.0109 (8)	0.0064 (9)	0.0039 (8)
C15	0.0353 (11)	0.0259 (10)	0.0277 (10)	0.0111 (9)	0.0142 (9)	0.0095 (8)
C16	0.0241 (9)	0.0216 (9)	0.0241 (9)	0.0066 (7)	0.0117 (8)	0.0030 (7)
C17	0.0209 (9)	0.0218 (9)	0.0190 (9)	0.0085 (7)	0.0102 (7)	0.0037 (7)
C18	0.0212 (9)	0.0256 (10)	0.0227 (9)	0.0069 (8)	0.0110 (8)	0.0032 (8)
C19	0.0279 (10)	0.0301 (10)	0.0280 (10)	0.0118 (8)	0.0189 (9)	0.0085 (8)
C20	0.0364 (11)	0.0310 (11)	0.0209 (9)	0.0161 (9)	0.0152 (9)	0.0036 (8)
C21	0.0316 (11)	0.0297 (11)	0.0224 (10)	0.0047 (9)	0.0101 (9)	−0.0031 (8)
C22	0.0249 (10)	0.0260 (10)	0.0252 (10)	0.0054 (8)	0.0125 (8)	0.0031 (8)
C23	0.0161 (8)	0.0206 (9)	0.0256 (9)	0.0056 (7)	0.0106 (7)	0.0022 (7)
C24	0.0260 (10)	0.0243 (10)	0.0256 (10)	0.0096 (8)	0.0137 (8)	0.0059 (8)
C25	0.0375 (11)	0.0295 (11)	0.0309 (11)	0.0131 (9)	0.0201 (9)	0.0014 (9)
C26	0.0394 (12)	0.0265 (11)	0.0409 (12)	0.0191 (9)	0.0222 (10)	0.0057 (9)
C27	0.0426 (12)	0.0340 (12)	0.0328 (11)	0.0235 (10)	0.0181 (10)	0.0130 (9)
C28	0.0298 (10)	0.0314 (11)	0.0247 (10)	0.0153 (9)	0.0135 (9)	0.0061 (8)
C29	0.0201 (9)	0.0192 (9)	0.0229 (9)	0.0057 (7)	0.0088 (8)	0.0027 (7)
C30	0.0216 (9)	0.0239 (10)	0.0313 (11)	0.0069 (8)	0.0115 (8)	0.0014 (8)
C31	0.0202 (10)	0.0261 (10)	0.0371 (12)	0.0051 (8)	0.0052 (9)	0.0020 (9)
C32	0.0346 (12)	0.0313 (12)	0.0289 (11)	0.0108 (9)	−0.0008 (9)	−0.0046 (9)
C33	0.0420 (13)	0.0459 (14)	0.0286 (11)	0.0206 (11)	0.0106 (10)	−0.0062 (10)
C34	0.0280 (10)	0.0387 (12)	0.0284 (11)	0.0161 (9)	0.0117 (9)	0.0016 (9)
C35	0.0229 (9)	0.0225 (9)	0.0261 (9)	0.0127 (7)	0.0152 (8)	0.0071 (7)
C36	0.0272 (10)	0.0320 (11)	0.0274 (10)	0.0147 (9)	0.0114 (9)	0.0074 (8)
C37	0.0454 (13)	0.0540 (15)	0.0277 (11)	0.0342 (12)	0.0185 (10)	0.0166 (10)
C38	0.0603 (16)	0.0481 (14)	0.0478 (14)	0.0406 (13)	0.0405 (13)	0.0326 (12)
C39	0.0465 (13)	0.0265 (11)	0.0590 (15)	0.0204 (10)	0.0389 (12)	0.0219 (10)
C40	0.0298 (10)	0.0218 (9)	0.0348 (11)	0.0109 (8)	0.0199 (9)	0.0061 (8)

### Geometric parameters (Å, °)

**Table:**

Pd—C1	1.9985 (18)	C18—C19	1.385 (3)
Pd—P2	2.3232 (5)	C18—H18	0.9500
Pd—P1	2.3393 (5)	C19—C20	1.385 (3)
Pd—Br	2.5353 (3)	C19—H19	0.9500
P1—C5	1.8248 (18)	C20—C21	1.381 (3)
P1—C17	1.8255 (18)	C20—H20	0.9500
P1—C11	1.8267 (18)	C21—C22	1.390 (3)
P2—C35	1.8188 (19)	C21—H21	0.9500
P2—C29	1.8247 (19)	C22—H22	0.9500
P2—C23	1.8363 (19)	C23—C28	1.393 (3)
N1—C1	1.330 (2)	C23—C24	1.397 (3)
N1—C2	1.337 (3)	C24—C25	1.386 (3)
N2—C1	1.332 (2)	C24—H24	0.9500
N2—C4	1.340 (3)	C25—C26	1.377 (3)
C2—C3	1.373 (3)	C25—H25	0.9500
C2—H2	0.9500	C26—C27	1.380 (3)
C3—C4	1.373 (3)	C26—H26	0.9500
C3—H3	0.9500	C27—C28	1.388 (3)
C4—H4	0.9500	C27—H27	0.9500
C5—C10	1.391 (3)	C28—H28	0.9500
C5—C6	1.394 (3)	C29—C34	1.390 (3)
C6—C7	1.385 (3)	C29—C30	1.397 (3)
C6—H6	0.9500	C30—C31	1.387 (3)
C7—C8	1.384 (3)	C30—H30	0.9500
C7—H7	0.9500	C31—C32	1.378 (3)
C8—C9	1.382 (3)	C31—H31	0.9500
C8—H8	0.9500	C32—C33	1.383 (3)
C9—C10	1.386 (3)	C32—H32	0.9500
C9—H9	0.9500	C33—C34	1.385 (3)
C10—H10	0.9500	C33—H33	0.9500
C11—C16	1.389 (3)	C34—H34	0.9500
C11—C12	1.398 (2)	C35—C40	1.389 (3)
C12—C13	1.386 (3)	C35—C36	1.400 (3)
C12—H12	0.9500	C36—C37	1.384 (3)
C13—C14	1.386 (3)	C36—H36	0.9500
C13—H13	0.9500	C37—C38	1.382 (4)
C14—C15	1.379 (3)	C37—H37	0.9500
C14—H14	0.9500	C38—C39	1.378 (4)
C15—C16	1.389 (3)	C38—H38	0.9500
C15—H15	0.9500	C39—C40	1.391 (3)
C16—H16	0.9500	C39—H39	0.9500
C17—C22	1.389 (3)	C40—H40	0.9500
C17—C18	1.400 (2)		
			
C1—Pd—P2	86.86 (5)	C18—C17—P1	120.85 (14)
C1—Pd—P1	90.58 (5)	C19—C18—C17	120.29 (18)
P2—Pd—P1	176.743 (17)	C19—C18—H18	119.9
C1—Pd—Br	176.56 (5)	C17—C18—H18	119.9
P2—Pd—Br	89.758 (13)	C20—C19—C18	119.93 (18)
P1—Pd—Br	92.815 (13)	C20—C19—H19	120.0
C5—P1—C17	102.50 (8)	C18—C19—H19	120.0
C5—P1—C11	103.18 (8)	C21—C20—C19	120.22 (18)
C17—P1—C11	103.89 (8)	C21—C20—H20	119.9
C5—P1—Pd	117.16 (6)	C19—C20—H20	119.9
C17—P1—Pd	112.70 (6)	C20—C21—C22	120.16 (19)
C11—P1—Pd	115.70 (6)	C20—C21—H21	119.9
C35—P2—C29	106.74 (9)	C22—C21—H21	119.9
C35—P2—C23	103.18 (8)	C17—C22—C21	120.16 (18)
C29—P2—C23	102.10 (8)	C17—C22—H22	119.9
C35—P2—Pd	110.52 (6)	C21—C22—H22	119.9
C29—P2—Pd	113.50 (6)	C28—C23—C24	118.21 (17)
C23—P2—Pd	119.58 (6)	C28—C23—P2	118.41 (14)
C1—N1—C2	115.99 (18)	C24—C23—P2	123.35 (14)
C1—N2—C4	116.48 (17)	C25—C24—C23	120.97 (18)
N1—C1—N2	125.98 (17)	C25—C24—H24	119.5
N1—C1—Pd	116.27 (13)	C23—C24—H24	119.5
N2—C1—Pd	117.66 (14)	C26—C25—C24	119.89 (19)
N1—C2—C3	122.9 (2)	C26—C25—H25	120.1
N1—C2—H2	118.6	C24—C25—H25	120.1
C3—C2—H2	118.6	C25—C26—C27	120.13 (19)
C2—C3—C4	116.5 (2)	C25—C26—H26	119.9
C2—C3—H3	121.7	C27—C26—H26	119.9
C4—C3—H3	121.7	C26—C27—C28	120.1 (2)
N2—C4—C3	122.2 (2)	C26—C27—H27	119.9
N2—C4—H4	118.9	C28—C27—H27	119.9
C3—C4—H4	118.9	C27—C28—C23	120.64 (19)
C10—C5—C6	119.00 (17)	C27—C28—H28	119.7
C10—C5—P1	122.17 (14)	C23—C28—H28	119.7
C6—C5—P1	118.80 (14)	C34—C29—C30	119.03 (18)
C7—C6—C5	120.63 (19)	C34—C29—P2	120.58 (15)
C7—C6—H6	119.7	C30—C29—P2	120.39 (14)
C5—C6—H6	119.7	C31—C30—C29	120.22 (19)
C8—C7—C6	119.70 (19)	C31—C30—H30	119.9
C8—C7—H7	120.2	C29—C30—H30	119.9
C6—C7—H7	120.2	C32—C31—C30	120.2 (2)
C9—C8—C7	120.28 (19)	C32—C31—H31	119.9
C9—C8—H8	119.9	C30—C31—H31	119.9
C7—C8—H8	119.9	C31—C32—C33	120.0 (2)
C8—C9—C10	120.07 (19)	C31—C32—H32	120.0
C8—C9—H9	120.0	C33—C32—H32	120.0
C10—C9—H9	120.0	C32—C33—C34	120.3 (2)
C9—C10—C5	120.32 (18)	C32—C33—H33	119.9
C9—C10—H10	119.8	C34—C33—H33	119.9
C5—C10—H10	119.8	C33—C34—C29	120.29 (19)
C16—C11—C12	118.97 (17)	C33—C34—H34	119.9
C16—C11—P1	119.85 (14)	C29—C34—H34	119.9
C12—C11—P1	121.13 (14)	C40—C35—C36	118.88 (18)
C13—C12—C11	120.14 (18)	C40—C35—P2	123.01 (15)
C13—C12—H12	119.9	C36—C35—P2	118.08 (15)
C11—C12—H12	119.9	C37—C36—C35	120.3 (2)
C12—C13—C14	120.48 (18)	C37—C36—H36	119.8
C12—C13—H13	119.8	C35—C36—H36	119.8
C14—C13—H13	119.8	C38—C37—C36	120.3 (2)
C15—C14—C13	119.52 (18)	C38—C37—H37	119.9
C15—C14—H14	120.2	C36—C37—H37	119.9
C13—C14—H14	120.2	C39—C38—C37	119.7 (2)
C14—C15—C16	120.50 (19)	C39—C38—H38	120.1
C14—C15—H15	119.8	C37—C38—H38	120.1
C16—C15—H15	119.8	C38—C39—C40	120.6 (2)
C11—C16—C15	120.38 (18)	C38—C39—H39	119.7
C11—C16—H16	119.8	C40—C39—H39	119.7
C15—C16—H16	119.8	C35—C40—C39	120.1 (2)
C22—C17—C18	119.23 (17)	C35—C40—H40	120.0
C22—C17—P1	119.88 (14)	C39—C40—H40	120.0

## References

[bb1] Beeby, A., Bettington, S., Fairlamb, I. J. S., Goeta, A. E., Kapdi, A. R., Niemela, E. H. & Thompson, A. L. (2004). *New J. Chem.* **28**, 600–605.

[bb2] Bruker (2001). *SADABS* Bruker AXS Inc., Madison, Wisconsin, USA.

[bb3] Bruker (2007). *SMART* and *SAINT* Bruker AXS Inc., Madison, Wisconsin, USA.

[bb4] Cardin, D. J., Cetinkaya, B. & Lappert, M. F. (1972). *Chem. Rev.* **72**, 545–574.

[bb5] Chin, C. H., Yeo, S. L., Loh, Z. H., Vittal, J. J., Henderson, W. & Hor, T. S. A. (1988). *J. Chem. Soc. Dalton Trans.* pp. 3777–3784.

[bb6] Dobrzynski, E. D. & Angelici, R. J. (1975). *Inorg. Chem.* **14**, 1513–1518.

[bb7] Hong, F. U., Huang, Y. L., Chen, P. P. & Chang, Y. C. (2002). *J. Organomet. Chem.* **655**, 49–54.

[bb8] Sheldrick, G. M. (2008). *Acta Cryst.* A**64**, 112–122. 10.1107/S0108767307043930 18156677

[bb9] Steffen, A., Sladek, M. I., Braun, T., Neumann, B. & Stammler, H. G. (2005). *Organometallics*, **24**, 4057–4064.

[bb10] Yih, K. H. & Lee, G. H. (2008). *J. Chin. Chem. Soc.* **55**, 109–114.

[bb11] Yih, K. H., Wang, H. F., Huang, K. F., Kwan, C. C. & Lee, G. H. (2009). *J. Chin. Chem. Soc.* **56**, 718–724.

